# TAM pathway proteins as novel salivary biomarkers for periodontitis

**DOI:** 10.1002/jper.70021

**Published:** 2025-11-04

**Authors:** Karina Mendes, Ana T. P. C. Gomes, Dimitris N. Tatakis, Tiago Marques, Marla Pinto, Pedro C. Lopes, Maria J. Correia, Nuno Rosa

**Affiliations:** ^1^ Faculty of Dental Medicine (FMD), Center for Interdisciplinary Research in Health (CIIS), Universidade Católica Portuguesa Viseu Portugal; ^2^ Department of Periodontics School of Dental Medicine Case Western Reserve University Cleveland Ohio USA; ^3^ Department of Oral and Maxillofacial Surgery, Oral Medicine, and Periodontology, College of Dentistry University of Jordan Amman Jordan

**Keywords:** AXL receptor tyrosine kinase, biomarkers, Mer tyrosine kinase, periodontitis, receptor protein‐tyrosine kinases, saliva, smoking

## Abstract

**Background:**

The identification of molecular biomarkers that help clinicians in early diagnosis is a key focus of periodontal research. The major aim of this cross‐sectional observational study was to assess whether salivary TAM pathway protein levels have the potential to discriminate between periodontally healthy or gingivitis controls and periodontitis patients, including those with mild (I/II) or severe (III/IV) disease.

**Methods:**

Twenty‐five periodontally healthy, 24 gingivitis, and 51 periodontitis patients further stratified into mild (stages I/II, *n* = 25) or severe (stages III/IV, *n* = 26) periodontitis were included. Salivary levels of tyrosine‐protein kinase receptor UFO (AXL), TYRO3 protein tyrosine kinase (TYRO3), Proto‐oncogene tyrosine‐protein kinase MER (MERTK), and growth arrest‐specific protein 6 (GAS6) were quantified using a multiplex immunoassay approach.

**Results:**

Salivary AXL, TYRO3, MERTK, and GAS6 levels were significantly elevated in periodontitis compared to periodontally healthy and gingivitis patients. Similar results for each periodontitis severity subgroup compared to control groups (except for MERTK, which was significantly different only for stage III/IV) were obtained. Supporting these findings, AXL, TYRO3, and GAS6 were the most accurate in differentiating between periodontally healthy/gingivitis and periodontitis, including mild or severe periodontitis (area under the curve [AUC] ranging from 0.72 to 0.89). Overall, combining biomarkers enhanced the predictive value for identifying periodontitis, including mild and severe disease, compared to using individual biomarkers alone (AUC values between 0.81 and 0.91).

**Conclusion:**

Salivary TAM pathway markers show promise as a potential noninvasive diagnostic screening tool to distinguish between controls (healthy/gingivitis) and periodontitis, including mild or severe periodontitis.

**Plain language summary:**

Salivary TAM pathway biomarkers can distinguish between periodontally healthy/gingivitis and periodontitis patients, including mild or severe periodontitis.

## 1 INTRODUCTION

Among chronic inflammatory conditions, periodontitis is particularly widespread, impacting millions of people around the world and requiring continuous monitoring and care.^1^ The cause of periodontitis is oral biofilm dysbiosis, involving the presence of highly pathogenic bacteria that trigger an immune/inflammatory host response.^2^ An uncontrolled inflammatory response, along with contributing factors such as host genetics,^3^ smoking, and diabetes,^4^ may lead to the loss of supporting tooth structures such as gingiva, periodontal ligament, and alveolar bone.^5^ Unlike periodontitis, gingivitis is a reversible condition marked by gum inflammation without any bone or attachment loss. In susceptible individuals, gingivitis can evolve to periodontitis.^5^

Despite the high prevalence of periodontitis and its established association with many systemic chronic diseases, like diabetes,^6^ rheumatoid arthritis,^7^ and cardiovascular disease^8^ diagnosis of the condition still relies on cumbersome conventional diagnostic processes.^9^ This fact, along with the typically silent nature of periodontitis, until its later stages, likely explains the significant diagnostic delay^10^ recently reported for the disease. Therefore, identification of molecular biomarkers, especially in saliva, is an active area of research,^11^, ^12^ given the potential to offer clinicians a rapid, noninvasive, precise, and early diagnostic tool, and to support risk evaluation and informed decision‐making. In fact, the new classification of periodontal diseases opened the opportunity to include biomarkers to help in periodontal diseases case definition and classification by severity and complexity (“stage”) and progression risk (“grade”).^13^

Reports have shown that the TAM receptor tyrosine kinases (RTKs), a subfamily of RTKs with 3 members, TYRO3, AXL, and MERTK, affect immune regulatory and inflammatory processes and interact with Toll‐like receptors.^14^, ^15^ These 3 TAM receptor proteins have recently been shown to be expressed in healthy human masticatory mucosa.^16^ The TAM pathway proteins, which include the 3 aforementioned TAM receptors and their ligands (growth arrest‐specific 6 [GAS6] and protein S [PROS1]) have been implicated in chronic inflammatory systemic diseases, for example, rheumatoid arthritis.^17^ Furthermore, evidence emerged in the past few years indicating that the TAM pathway might be involved in the pathogenesis of periodontitis^18^, ^19^, ^20^ and periimplantitis.^21^ The TAM receptors are also found in soluble form (sTAM), acting as decoy receptors that inhibit TAM receptor activation.^22^ Altered sTAM levels, whether locally or systemically, have been correlated with disease severity for diseases such as rheumatoid arthritis^23^ and lupus nephritis.^24^ However, there are no studies on the possible association of sTAM with periodontal diseases. Therefore, the main goal of this study was to assess whether salivary levels of TAM receptors and GAS6, individually and/or in combination, are correlated with periodontal health status and whether they can discriminate between health and disease (gingivitis/periodontitis), as well as between control and mild (stages I/II) or severe (stages III/IV) periodontitis.

## 2 MATERIALS AND METHODS

### 2.1 Study design and study population

The present study was cross‐sectional observational, including patients accompanied in dental clinic appointments at the University Dental Clinic of the Universidade Católica Portuguesa. Patients were recruited if they met the eligibility criteria described below. The patients who accepted to integrate this study were notified about the protocol and procedures, and signed a written consent form and filled out a survey before any clinical intervention. Study included a single visit, and each patient contributed a single sample to the study. The Ethics Committee for Health of the Universidade Católica Portuguesa (CES‐UCP; protocol #CES/UCP‐157, 21/10/2021) approved this study protocol. The study was performed in conformity with the Helsinki Declaration of 1975 (revised in 2013) and adheres to the STROBE guidelines.

The inclusion criteria were; adults, aged 19–85 years old, and presence of at least 18 natural teeth. The following exclusion criteria were applied: individuals that received treatment for periodontitis within the past 6 months, pregnant women, individuals with diabetes mellitus, chronic obstructive pulmonary disease, autoimmune diseases, oncological diseases, inflammatory bowel disease, or other serious illnesses. Patients taking antibiotics, anti‐inflammatory drugs, and other medications that could affect periodontal tissues were excluded from this study.

### 2.2 Study procedures

#### 2.2.1 Clinical assessment

Periodontal examinations were performed by 2 experienced trained periodontists (TM, PCL), who recorded clinical measurements to the nearest millimeter, utilizing a North Carolina Probe 54B* periodontal probe, and rendered a periodontal diagnosis based on the Classification of Periodontal and Peri‐Implant Diseases and Condition^25^ criteria proposed by the 2017 World Workshop, which classifies patients as periodontally healthy, gingivitis, and periodontitis (stage and grade). Smoking status was determined through self‐reporting.

#### 2.2.2 Sample collection

To collect unstimulated whole saliva samples, the spitting was used according to formerly recognized protocols.^26^, ^27^ Briefly, saliva samples were collected using the passive drooling method into sterile 50 mL tubes without stabilizers or protease inhibitors. Saliva samples were immediately placed on ice, processed and stored at −80° C to carry out the immunoassays.

#### 2.2.3 Biochemical analysis

Saliva samples were taken from the freezer and centrifuged at 10,000 × *g* for 10 min at 4° C. Supernatant was recovered and used for TAM receptors (AXL, MERTK, TYRO3) and GAS6 protein quantification through multiplex bead‐based immunoassay technology. A customized kit† was used. All procedures were completed following the operating manual using a multiplex reader‡ for data acquisition, following methodology established in SalivaTec laboratory and previously detailed.^27^ Each salivary sample was analyzed in duplicate, and obtained values were averaged.

#### 2.2.4 Sample size and statistical analysis

The sample size and statistical power evaluation indicated that a minimum of 23 samples were required per group to achieve a 90% power to identify 0.40 f‐type effect size with a Type‐I error of 0.05.

For normally distributed data, descriptive statistics are presented as mean ± standard deviation, for non‐normally distributed data are given as median with interquartile range (IQR) and for the qualitative variables data is presented as frequencies or percentages. To check the normality of the continuous data the Shapiro–Wilk test was used. For categorical data, chi‐squared test or Fisher's exact test was utilized to determine the relationship between the qualitative variables among the groups under study. Regarding the statistical analysis of continuous variables, an Independent‐Samples *T* Test for normally distributed data and the Mann–Whitney *U* test for non‐normally distributed data were used. For differences comparison among groups the Kruskal–Wallis test was conducted. Post hoc pairwise comparisons were completed utilizing Dunn's test with Bonferroni correction for multiple comparisons.

To analyze differentiation between groups, area under curve (AUC) and receiver‐operating characteristics (ROC) studies for the TAM biomarkers were conducted. To choose the ideal cutoff point (threshold) for a biomarker, estimation of the Youden index (*J*) method^28^ was performed. For biomarker combination, logistic regression analysis was used. The chance of incorrectly rejecting a true null hypothesis was set up to *p* ≤ 0.05.

Statistical analyses and graphic presentations were executed using commercially available statistical§ and scientific graphing software.‖

## 3 RESULTS

### 3.1 Study population

The study recruited 100 participants distributed into 3 groups: periodontal health (*n* = 25), gingivitis (*n* = 24), and periodontitis (*n* = 51), whose detailed demographics are reported in Table 1. Noticeable age difference across the different patient groups (*p* < 0.0001), with the periodontitis group showing the highest median age compared to the other 2 groups. Overall, there were few current smokers in the study population (4%), all of them in the periodontitis group. Statistical analysis revealed a significant relationship between smoking status and periodontitis (*p* = 0.03).

**TABLE 1:** Study population characteristics.

Parameter	Healthy (*n* = 25)	Gingivitis (*n* = 24)	Periodontitis (*n* = 51)	*p*‐value
Age (years)	28 (25.5)	25.50 (12.25)	56 (22)^b^	<0.0001^a^
Sex (F/M)	14/11	16/8	20/31	0.0672^c^
Smoking status, *n* (%)				
Current smoker	0	0	4 (7.8)	0.03^c^
Former smoker	0	0	6 (11.8)	
Never smoker	25 (100)	24 (100)	41 (80.4)	

*Notes*: Age values are reported as median with interquartile range (IQR).

^a^ Kruskal–Wallis test with Bonferroni correction.

^b^ Healthy versus Periodontitis and Gingivitis versus Periodontitis (*p* < 0.0001); Mann–Whitney *U* test.

^c^ Chi‐squared test.

Participants in the periodontitis group were further classified as having mild (stages I/II, *n* = 25) or severe (stages III/IV, *n* = 26) disease, with more detailed demographics shown in Table 2. Age was significantly different between mild and severe periodontitis (*p* = 0.0038) with a higher mean age (62.1 ± 14.5) observed in patients at stages III/IV. Similarly, a significant association between smoking status and periodontitis stage was also detected (*p* = 0.02). Most patients were classified as grade B periodontitis and statistical analysis revealed a significant association between periodontitis grade and stage (*p* = 0.03).

**TABLE 2:** Periodontitis patient classification and demographics.

Parameter	Stages I/II (*n* = 25)	Stages III/IV (*n* = 26)	*p*‐value
Age (years)	49.8 (14.3)	62.1 (14.5)	0.0038^a^
Sex (F/M)	9/16	11/15	0.7761^b^
Smoking status, *n* (%)			
Current smoker	0 (0)	4 (15.4)	0.02^c^
Former smoker	1 (4)	5 (19.2)	
Never smoker	24 (96)	17 (65.4)	
Periodontitis grade, *n*			
A	1	0	0.03^c^
B	24	20	
C	0	6	

*Note*: Age values are reported as mean (SD).

^a^ Independent‐Samples *T* test.

^b^ Fisher's exact test.

^c^ Chi‐squared test.

### 3.2 Soluble TAM pathway protein quantification

**FIGURE 1:**
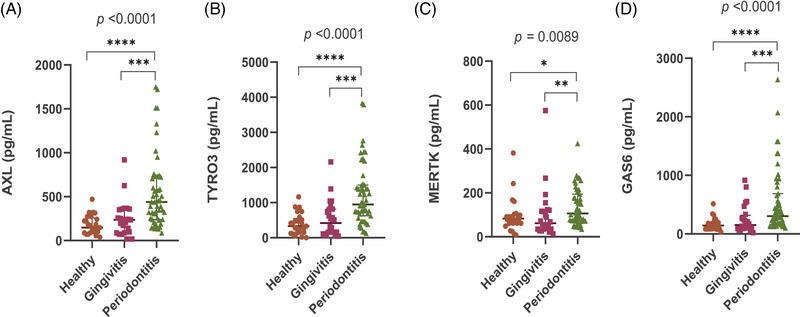
Quantification (pg/mL) of soluble TAM pathway proteins in saliva samples from patients with periodontal health (healthy), gingivitis and periodontitis. (A) AXL: Tyrosine‐protein kinase receptor UFO; (B) TYRO3: TYRO3 protein tyrosine kinase; (C) MERTK: Proto‐oncogene tyrosine‐protein kinase MER; (D) GAS6: Growth arrest‐specific protein 6. Data is presented as median with interquartile range (IQR). Statistical analysis with p value derived from the Kruskal–Wallis test and Bonferroni test. To assess the statistical significance between 2 independent groups the Mann–Whitney *U* Test was used. *p ≤ 0.05; **p ≤ 0.01; ***p ≤ 0.001; ****p ≤ 0.0001.

All 100 patient samples were positive for AXL, TYRO3, MERTK, and GAS6. The data from the quantification of the salivary TAM pathway proteins across the 3 patient groups (periodontally healthy, gingivitis, and periodontitis) are depicted in Figure 1 and Table S1 in the online *Journal of Periodontology*. The data indicated statistically significant differences between groups for all 4 biomarkers. Specifically, the periodontitis group values were significantly higher than either the gingivitis or the health group values. Nevertheless, no significant difference between gingivitis and health groups for any of the biomarkers were obtained.

To evaluate the potential influence of smoking on salivary TAM protein levels, we compared concentrations among periodontitis patients classified as current (*n* = 4), former (*n* = 6), and never smokers (*n* = 41). No statistically significant differences in protein levels were observed between these subgroups (*p* ≥ 0.3841; Figure S1 in online *Journal of Periodontology*).

**FIGURE 2:**
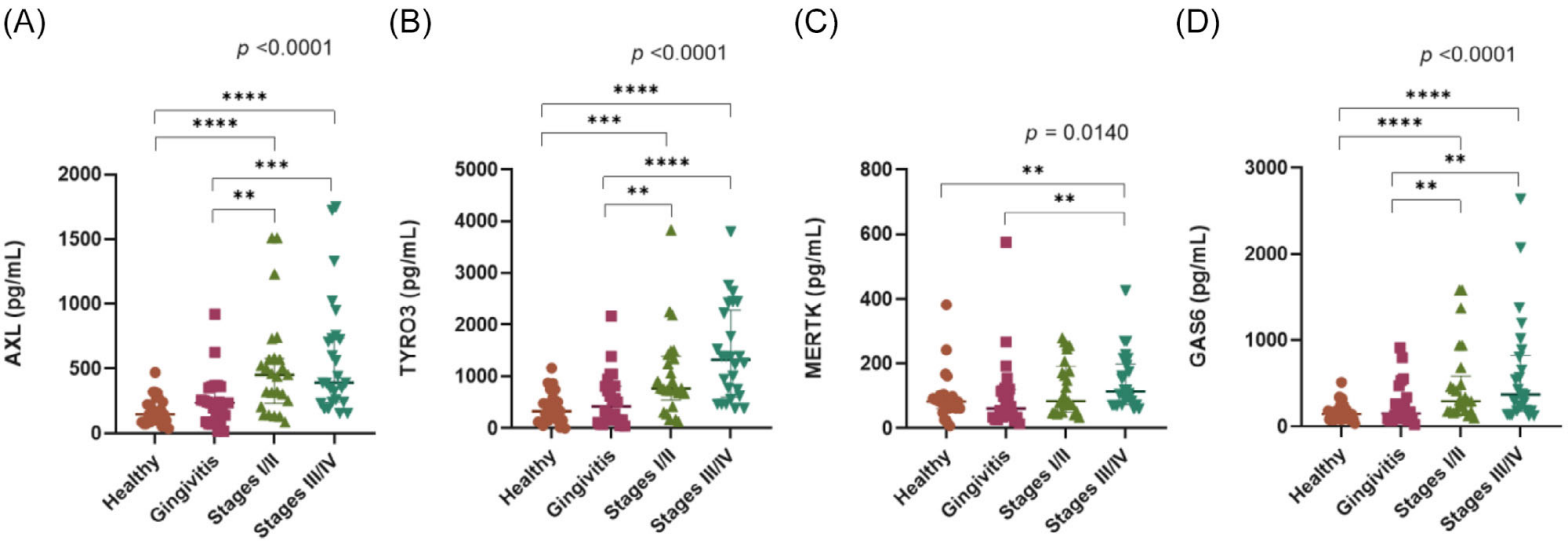
Quantification (pg/mL) of soluble TAM pathway proteins in saliva samples from patients with periodontal health (healthy), gingivitis, mild (stages I/II), and severe (stages III/IV) periodontitis. (A) AXL: Tyrosine‐protein kinase receptor UFO; (B) TYRO3: TYRO3 protein tyrosine kinase; (C) MERTK: Proto‐oncogene tyrosine‐protein kinase MER; (D) GAS6: Growth arrest‐specific protein 6. Data are presented as median with IQR. Statistical analysis with *p*‐value derived from the Kruskal–Wallis test and Bonferroni test. To assess the statistical significance between 2 independent groups the Mann–Whitney *U* Test was used. **p ≤ 0.01; ***p ≤ 0.001; ****p ≤ 0.0001. IQR, interquartile range.

Salivary TAM pathway protein levels were also compared based on periodontitis severity (Figure 2 and Table S2 in online *Journal of Periodontology*). Although the biomarker level differences between health/gingivitis and periodontitis groups remained significant for each periodontitis severity subgroup (except for MERTK, which was significantly different only for stage III/IV), differences between the 2 severity categories were not significant for any biomarker.

### 3.3 Diagnostic performance of TAM pathway biomarkers

**FIGURE 3:**
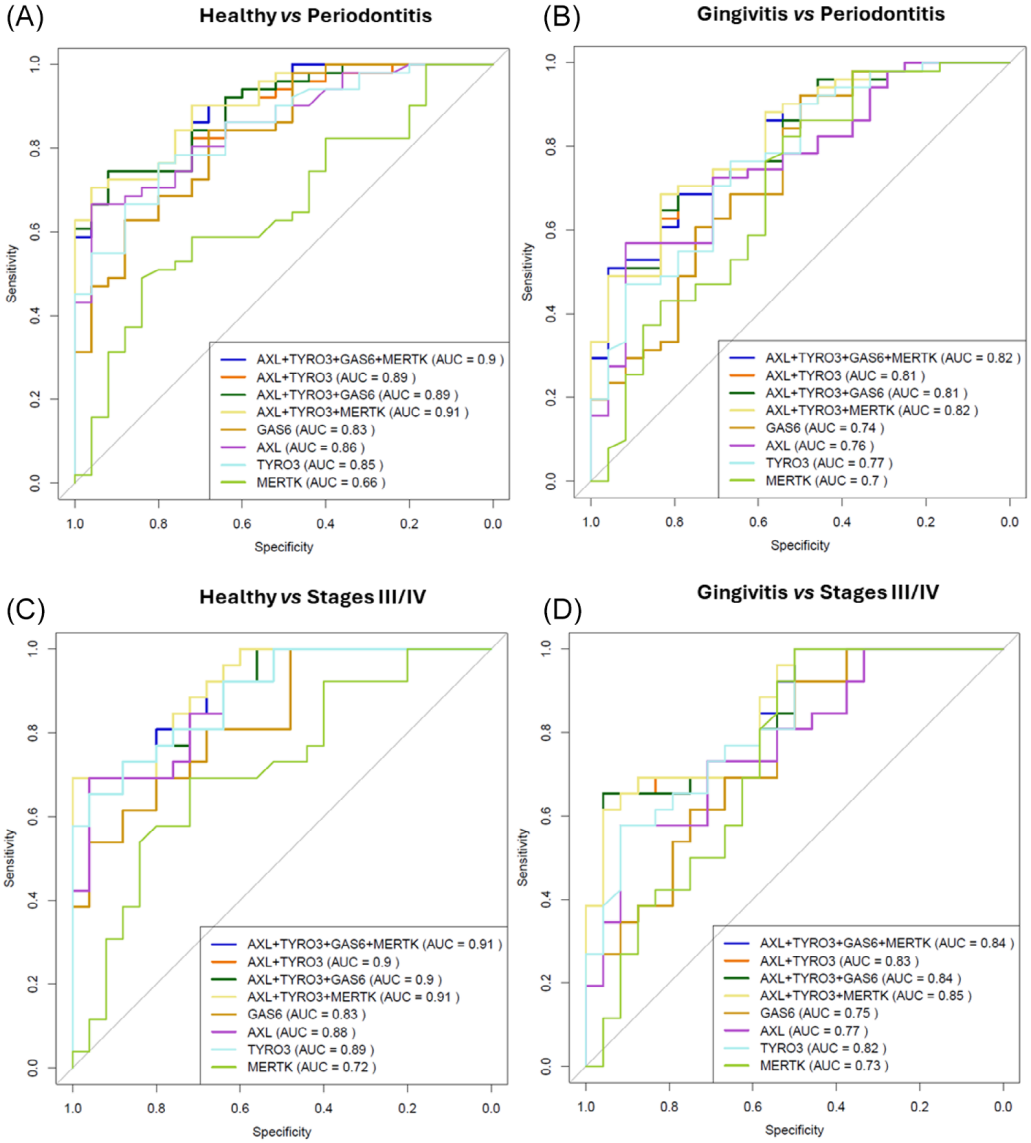
ROC curves and corresponding AUCs of single salivary TAM pathway proteins and some of their combinations tested for periodontitis screening. Discriminating capacity of salivary TAM biomarkers between periodontally healthy and periodontitis (A), gingivitis and periodontitis (B), periodontally healthy and stages III/IV (severe periodontitis) (C), gingivitis and stages III/IV (severe periodontitis) (D). Interpretation of the AUC^29^: 0.9 ≤ AUC (excellent); 0.8 ≤ AUC < 0.9 (good); 0.7 ≤ AUC < 0.8 (fair); 0.6 ≤ AUC < 0.7 (poor); 0.5 ≤ AUC < 0.6 (fail). AUC, area under the curve; ROC, receiver‐operating characteristic.

To assess the overall diagnostic performance of the analyzed biomarkers, ROC curves were generated, and their corresponding area under the curve (AUC)^29^ values are presented in Figure 3 and Figure S2 in the online *Journal of Periodontology*. Additional metrics, including cutoff points, sensitivity, specificity, negative predictive value (NPV), and positive predictive value (PPV), are detailed in Table 3 for comparisons between periodontal health and periodontitis (which showed the highest AUC values). Tables S3–S7 in the online *Journal of Periodontology* provide these metrics for comparisons involving the remaining patient groups.

**TABLE 3:** Screening efficacy of individual and combined salivary TAM biomarkers between periodontal health and periodontitis.

Biomarker	Cutoff (pg/mL)	Sensitivity	Specificity	PPV	NPV	AUC (95% CI)
GAS6	172.66	0.84	0.68	0.84	0.68	0.83 (0.74–0.93)
AXL	321.44	0.67	0.96	0.97	0.59	0.86 (0.77–0.94)
TYRO3	606.06	0.76	0.80	0.89	0.63	0.85 (0.76–0.93)
MERTK	107.54	0.49	0.84	0.86	0.45	0.66 (0.53–0.79)
GAS6 + AXL + TYRO3 + MERTK		0.73	0.92	0.95	0.62	0.90 (0.84–0.97)
AXL + TYRO3		0.71	0.96	0.97	0.62	0.89 (0.82–0.96)
AXL + GAS6		0.63	0.96	0.97	0.56	0.87 (0.79–0.95)
AXL + MERTK		0.67	0.96	0.97	0.59	0.86 (0.77–0.94)
TYRO3 + GAS6		0.67	0.96	0.97	0.59	0.87 (0.79–0.95)
TYRO3 + MERTK		0.78	0.80	0.89	0.65	0.84 (0.76–0.93)
MERTK + GAS6		0.65	0.88	0.92	0.55	0.83 (0.74–0.93)
AXL + TYRO3 + GAS6		0.75	0.92	0.95	0.64	0.89 (0.83–0.96)
AXL + TYRO3 + MERTK		0.71	0.96	0.97	0.62	0.91 (0.84–0.97)
AXL + GAS6 + MERTK		0.71	0.92	0.95	0.61	0.87 (0.79–0.95)
TYRO3 + GAS6 + MERTK		0.67	0.96	0.97	0.59	0.86 (0.78–0.94)

Abbreviations: AUC, area under the curve; CI, confidence interval; NPV, negative predictive value; PPV, positive predictive value.

Three of the biomarkers, AXL, TYRO3 and GAS6 were the most accurate in differentiating between periodontal health and periodontitis (AXL: sensitivity = 0.67, specificity = 0.96, cutoff value 321.44 mg/mL; TYRO3: sensitivity = 0.76, specificity = 0.80; cutoff value 606.06 pg/mL; GAS6: sensitivity = 0.84, specificity = 0.68; cutoff value 172.66 pg/mL; AUC ranging from 0.83 to 0.86), including mild (stages I/II) or severe (stages III/IV) periodontitis. However, the potential of AXL, TYRO3, MERTK, and GAS6 to discriminate between gingivitis and periodontitis is moderate, as indicated by their AUC values varying between 0.7 and 0.77. Interestingly, TYRO3 shows promising potential in differentiating between gingivitis and severe periodontitis (stages III/IV) as reflected by its AUC (0.82).

ROC curves for biomarker combinations across all group comparisons were also generated. Although in Figure 3 and Figure S2 only the ROC curves yielding the highest AUC values (0.78 to 0.91) are presented, the Tables S3–S7 provide detailed metrics for all possible biomarker combinations. Overall, combining biomarkers may enhance the predictive value for identifying periodontitis, including its mild and severe stages, compared to using individual biomarkers alone. Moreover, biomarker combinations seem to be more accurate in differentiating between periodontitis and periodontal health (AUC values between 0.89 and 0.91) than discriminating between periodontitis and gingivitis (AUC values between 0.81 and 0.82).

## 4 DISCUSSION

The main goal of this work was to investigate the salivary levels of TAM pathway proteins, that is, the 3 TAM receptors (AXL, TYRO3, and MERTK) and GAS6, in periodontal health, gingivitis, and periodontitis, and to assess their ability to discriminate between health and disease. The results of the study, which is the first of its kind, indicate that all 4 TAM pathway proteins are readily detectable in human saliva, regardless of periodontal health status, and that the salivary levels of these biomarkers could help distinguish between controls (health, gingivitis) and periodontitis, regardless of the severity of periodontitis (stage I/II or stage III/IV). These novel findings suggest that analysis of the salivary levels of TAM pathway proteins could be a promising noninvasive tool to rapidly diagnose and differentiate between periodontitis, both mild and severe, from periodontal health and gingivitis.

Among the various pathogenetic mechanisms implicated in periodontitis,^2^, ^5^ the TAM receptor tyrosine kinase pathway is a more recently implicated one, as demonstrated from results of in vivo models and in vitro studies.^18^, ^19^ Our findings also indicate that the consistent detection of salivary TAM biomarkers in health and periodontitis mirrors the reported detection of mRNA expression of TAM proteins in gingival tissues of both healthy and periodontitis patients^18^, ^19^; there are no reports on gingival mRNA expression of these proteins in gingivitis patients. The TAM family includes 3 receptor tyrosine kinases—TYRO3, AXL, and MERTK, which are primarily expressed in dendritic cells, macrophages, and natural killer cells, and in non‐immune cells, such as epithelial cells.^30^ TAM receptors along with Gas6 and Protein S ligands, have a critical function in the termination of inflammation by inhibiting pro‐inflammatory and enhancing anti‐inflammatory cytokine production and modulating immune cell activation, among other mechanisms.^22^ The TAM receptors are also found in a soluble form, acting as decoy receptors that inhibit TAM receptor activation.^22^ Dysregulation of soluble TAM (sTAM) receptor levels is associated with distinct inflammatory and autoimmune diseases such as rheumatoid arthritis,^23^ lupus nephritis^24^ and primary Sjögren's syndrome,^31^ and a correlation between sTAM receptors and disease severity has been reported.

Consistent with the association between systemic chronic diseases and sTAM, salivary sTAM levels were strongly associated with periodontitis as demonstrated by our findings. The elevated salivary sTAM levels in periodontitis appear to contradict the reported similar AXL, GAS6 and MERTK mRNA expression in healthy and periodontitis patient gingiva.^18^, ^19^ However, this inconsistency can be linked to the fact that transcriptional (mRNA) changes do not always correlate with translational (protein) changes^32^ due to variances in the overall protein production process, such as mRNA and protein stability; alternatively, a potentially more diverse tissue origin of the salivary sTAM proteins might explain the discrepancy. The lack of prior studies on TAM pathway proteins in gingivitis precludes any comparisons.

All sTAM biomarkers analyzed were detectable and showed differences between health and disease. However, MERTK protein levels were the most discrepant, being significantly different from control groups only when compared to severe periodontitis patients (stages III/IV). This finding may suggest a differential salivary shedding efficiency of MERTK and/or true differences in soluble MERTK levels depending on disease context and severity. In fact, studies have reported varying levels of sTAM receptors in biofluids, such as plasma and synovial fluid, from patients with lupus nephritis^24^ and rheumatoid arthritis,^23^ respectively.

Although no previous studies have evaluated salivary TAM protein levels, the present study ROC curve analyses demonstrated that these proteins, individually or in combination, exhibit diagnostic accuracy comparable to or even superior to well‐established salivary periodontitis biomarkers, such as interleukin (IL) ‐1β,^33^, ^34^, ^35^ IL‐6,^35^, ^36^ MMP‐8, and MMP‐9.^34^, ^37^, ^38^ For example, the reported AUC for IL‐1β in distinguishing periodontitis from healthy subjects (0.88),^33^ is similar to the 0.83–0.86 AUC found for AXL, TYRO3, and GAS6; however, IL‐1β showed a lower AUC (0.66) for differentiating gingivitis from periodontitis compared to the sTAM AUCs (0.70–0.77). Combining IL‐1β with other biomarkers improved diagnostic performance (AUC = 0.94 and 0.77 for periodontitis vs. healthy and for periodontitis vs. gingivitis, respectively),^33^ mirroring the present study findings for sTAM protein combinations (AUC = 0.90 and 0.82, respectively). Overall, the results reported herein align with previous studies,^33^, ^39^, ^40^ demonstrating that salivary biomarker combinations, rather than individual biomarkers, enhance diagnostic accuracy, yielding higher AUC values across all group comparisons.

Unlike other studies,^41^, ^42^ the current work included 2 control groups, healthy and gingivitis, for comparisons with periodontitis group and subgroups (mild/severe periodontitis). Although sTAM proteins effectively distinguished periodontitis and subgroups from controls, the diagnostic accuracy was lower when using gingivitis as control, as also observed by other authors.^33^ These findings might reflect overlapping inflammatory and immune responses in gingivitis and early periodontitis.^43^

Elevated TAM receptor expression has been reported in monocytes isolated from peripheral blood from older people.^44^ Given the age difference among patient groups in the present study, it would be conceivable to ascribe the identified TAM protein level increases to age‐related changes. However, the study on peripheral blood monocytes^44^ measured expression of transmembrane receptors instead of soluble proteins, was restricted to healthy volunteers, and the difference in average age between the 2 groups (younger: 26 years; older: 74 years) was far greater than the age difference between periodontitis patients (especially stage I/II) and controls in the current study. In the context of periodontitis, the observed differences in sTAM levels are likely to be related to inflammation and tissue destruction rather than age‐related changes. Consistent with this premise, the expression of pro‐inflammatory cytokines such as IL‐1β^34^, ^42^ and IL‐6,^36^, ^42^ reliable periodontitis indicators, tends to increase with age^45^, ^46^; however, their elevated levels in periodontitis patients are primarily driven by disease severity rather than age.^35^, ^36^

There were no significant differences in sTAM protein levels among periodontitis patients with different smoking status, suggesting that smoking has no effect on the salivary TAM protein levels. This aligns with previous research showing no difference in TYRO3 levels between heavy smokers and non‐smokers in human spermatozoa,^47^ though lower GAS6 levels have been reported in smokers with psoriasis.^48^ These mixed findings in the literature underscore that the influence of smoking on TAM protein expression may be disease‐ and/or tissue‐specific. In the present study, the lack of significant smoking‐related variation within the periodontitis group strengthens the interpretation that the observed differences in TAM levels are primarily disease‐related.

The main limitation of our study is the absence of ethnic diversity in the study population, which may restrict the generalizability of the findings. Since genetic and environmental factors can influence biomarker expression and disease susceptibility,^34^, ^42^ further studies including racially diverse populations are necessary to validate the present results and assess potential variations in biomarker levels and disease stage among ethnic groups. The uneven distribution of smoking status, that is, the fact that current smokers, albeit comprising only 8% of the entire periodontitis group, were all concentrated in the severe periodontitis subgroup (stage III/IV), is another study limitation. While subgroup analysis suggested that smoking had no significant impact on salivary TAM protein levels, the overlap between smoking and disease severity introduces potential residual confounding. Future studies with larger sample sizes and more balanced smoking representation among healthy and gingivitis patients are warranted to further disentangle the possible effects of smoking on salivary sTAM levels. Lastly, PROS1 (TAM ligand) was not quantified in this study due to limitations of the multiplex immunoassay platform used, which did not support simultaneous measurement of this analyte. However, given its relevance in the TAM inflammatory pathway, future studies should consider quantifying this protein individually or through alternative immuno‐based approaches. Strengths of the present study include the employment of the latest periodontal disease classification, the stratification of the periodontitis group into mild and severe disease, and the inclusion of 2 control groups, periodontally healthy individuals and gingivitis patients.

Additional studies are required to assess the potential of salivary sTAM biomarkers in monitoring treatment efficacy. Moreover, gaining more insight into the mechanisms by which sTAM receptors might be contributing to periodontitis pathogenesis could allow for the advancement in innovative treatment plans aimed at modulating the influence of the TAM pathway on the disease.

## 5 CONCLUSION

Within the study limitations, salivary sTAM proteins are promising noninvasive screening biomarkers for periodontitis. AXL, TYRO3, and GAS6 were the most precise biomarkers in distinguishing periodontitis from periodontal health and gingivitis, and biomarker combinations had a stronger predictive value than single biomarkers.

## AUTHOR CONTRIBUTIONS

Karina Mendes contributed to study conception and design; direction of research implementation; data acquisition, analysis, presentation and interpretation; and drafting, editing, and revising the manuscript. Ana T. P. C. Gomes contributed to study conception and design; data acquisition, analysis, and interpretation; and drafting, editing, and revising the manuscript. Dimitris N. Tatakis contributed to study design, data interpretation, and drafting, editing, and revising the manuscript. Tiago Marques contributed to sample collection, data interpretation, and editing and revising the manuscript. Marla Pinto contributed to data acquisition and editing and revising the manuscript. Pedro C. Lopes contributed to sample collection and editing and revising the manuscript. Maria J. Correia contributed to data interpretation and editing and revising the manuscript. Nuno Rosa contributed to funding acquisition, data interpretation, and editing and revising the manuscript. All authors reviewed and approved the submitted manuscript.

## CONFLICT OF INTEREST STATEMENT

The authors declare no conflicts of interest.

## Supporting information



Supporting Information

## Data Availability

The data that support the findings of this study are available from the corresponding author upon reasonable request.

## Footnotes

* LM‐Dental, LM‐Instruments Oy, Parainen, Finland

† ProcartaPlex immunoassay ‐ 4 Plex Panel Kit; Thermo Fisher Scientific Inc., Waltham, Massachusetts, USA

‡ Bio‐Plex MAGPIX Multiplex Reader; Bio‐Rad Laboratories Inc., Hercules, California, USA

§ R version 4.4.1; R Foundation for Statistical Computing, Vienna, Austria

‖ GraphPad Prism 8; GraphPad Software, LLC, Boston, Massachusetts, USA
