# Supplementary material for: TAM pathway proteins as novel salivary biomarkers for periodontitis
Source: J Periodontol. 2025 Nov 4;97(4):760–70. doi: 10.1002/jper.70021 (PMC13169470; doi:10.1002/jper.70021)
Supplement: Supplementary file 1 — Supporting Information [file JPER-97-760-s001.pdf]

TAM pathway proteins as novel salivary biomarkers for periodontitis

Karina **Mendes**[*], Ana T. P. C. **Gomes**[*], Dimitris N. **Tatakis**[†‡], Tiago **Marques**[*], Marla **Pinto**[*], Pedro C. **Lopes**[*], Maria J. **Correia**[*], Nuno **Rosa**[*]

[*]Universidade Católica Portuguesa,Faculty of Dental Medicine (FMD), Center for Interdisciplinary Research in Health (CIIS), Viseu, Portugal
[†]Department of Periodontics, School of Dental Medicine, Case Western Reserve University,Cleveland, Ohio, USA
[‡]Department of Oral and Maxillofacial Surgery, Oral Medicine, and Periodontology, College of Dentistry, University of Jordan, Amman, Jordan

---

SUPPLEMENTARY INFORMATION

SUPPLEMENTARY FIGURES

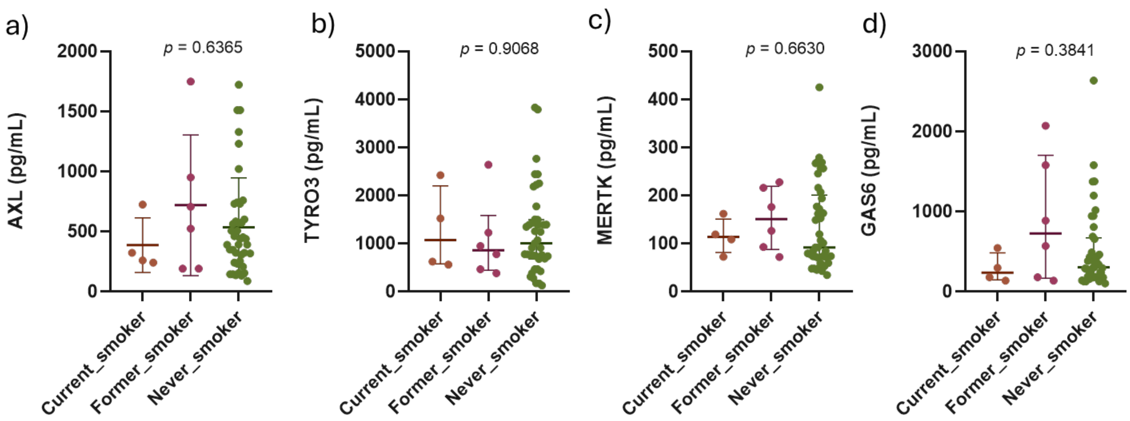

Supplementary Figure S1. Quantification (pg/mL) of soluble TAM pathway proteins in saliva samples from patients with periodontitis according to smoking status (current, former, and never smoker). a) AXL: Tyrosine-protein kinase receptor UFO; b) TYRO3: TYRO3 Protein Tyrosine Kinase; c) MERTK: Proto-oncogene tyrosine-protein kinase MER; d) GAS6: Growth arrest-specific protein 6. Data is presented as median with interquartile range (IQR). Statistical analysis with *p* value derived from the Kruskal-Wallis test and Bonferroni test. To assess the statistical significance between two independent groups the Mann-Whitney U Test was used. Analysis showed no statistically significant differences between groups ($p \geq 0.3841$).

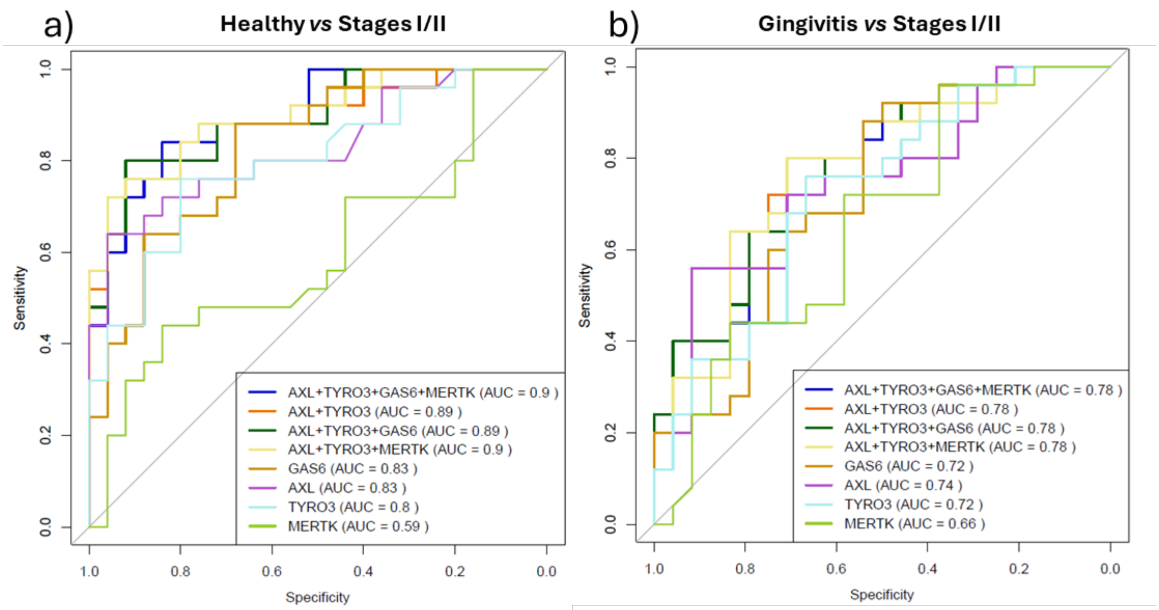

Supplementary Figure S2. Receiver-operating characteristic (ROC) curves and corresponding AUCs of single salivary TAM pathway proteins and some of their combinations tested for periodontitis screening. Discriminating capacity of salivary TAM biomarkers between periodontally healthy and stages I/II (mild periodontitis) (a), gingivitis and stages I/II (mild periodontitis) (b). Interpretation of the AUC[28]: $0.9 \leq$ AUC (excellent); $0.8 \leq$ AUC < 0.9 (good); $0.7 \leq$ AUC < 0.8 (fair); $0.6 \leq$ AUC < 0.7 (poor); $0.5 \leq$ AUC < 0.6 (fail). AUC, area under the curve

SUPPLEMENTARY TABLES

Supplementary table S1. AXL, TYRO3, MERTK and GAS6 salivary levels in Periodontally Healthy (H), Gingivitis (G) and Periodontitis (P) patients

	**H (n = 25)**	**G (n = 24)**	**P (n = 51)**	**Overall *p***	**H vs G *p***	**H vs P *p***	**G vs P *p***
**AXL (pg/mL)**	147.0 (163.6)	235.7 (267.2)	438.5 (483.3)	<0.0001	0.2504	<0.0001	0.0002
**TYRO3 (pg/mL)**	329.7 (398.8)	418.9 (656.5)	945.9 (882)	<0.0001	0.4657	<0.0001	0.0001
**MERTK (pg/mL)**	83.3 (39.3)	61.6 (85.1)	106.4 (121.8)	0.0089	0.6734	0.0273	0.0056
**GAS6 (pg/mL)**	144.8 (110.8)	150.2 (222.8)	303.4 (508.5)	<0.0001	0.2939	<0.0001	0.0007

H: Periodontally Healthy; G: Gingivitis; P: Periodontitis;
Data is presented as median with interquartile range (IQR).
Overall *p* value derived from the Kruskal-Wallis test and Bonferroni test.
Mann-Whitney U test used to assess differences between two independent groups (p value).

Supplementary table S2. AXL, TYRO3, MERTK and GAS6 salivary levels in Periodontally Healthy (H), Gingivitis (G) and Periodontitis I/II - III/IV patients

	**H (n = 25)**	**G (n = 24)**	**I/II (n = 25)**	**III/IV (n=26)**	**Overall *p***	**H *vs* G *p***	**H *vs* I/II *p***	**H *vs* III/IV *p***	**G *vs* I/II *p***	**G *vs* III/IV *p***	**I/II *vs* III/IV *p***
**AXL (pg/mL)**	147.0 (163.6)	235.7 (267.2)	452.3 (348.9)	390.0 (496.4)	<0.0001	0.2504	<0.0001	<0.0001	0.0030	0.0007	0.5465
**TYRO3 (pg/mL)**	329.7 (398.8)	418.9 (656.5)	773.8 (837.1)	1321 (1665.6)	<0.0001	0.4657	0.0002	<0.0001	0.0071	<0.0001	0.0838
**MERTK (pg/mL)**	83.3 (39.3)	61.6 (85.1)	84.40 (140.5)	113.9 (127)	0.0140	0.6734	0.2567	0.0076	0.0589	0.0037	0.3564
**GAS6 (pg/mL)**	144.8 (110.8)	150.2 (222.8)	293.1 (411.5)	372.1 (648.5)	<0.0001	0.2939	<0.0001	<0.0001	0.0062	0.0020	0.6843

H: Periodontally Healthy; G: Gingivitis; I/II: Stages I/II; III/IV: Stages III/IV.
Data is presented as median with interquartile range (IQR).
Overall *p* value derived from the Kruskal-Wallis test and Bonferroni test.
Mann-Whitney U Test to assess differences between two independent groups (*p* value).

Supplementary table S3. Screening efficacy of individual and combined salivary TAM biomarkers between gingivitis and periodontitis.

Biomarker	Cut off (pg/mL)	Sensitivity	Specificity	PPV	NPV	AUC (95% CI)
**GAS6**	134.08	0.92	0.50	0.80	0.75	0.74 (0.61-0.87)
**AXL**	378.04	0.57	0.92	0.94	0.50	0.76 (0.64-0.87)
**TYRO3**	621.76	0.76	0.67	0.83	0.57	0.77 (0.66-0.89)
**MERTK**	69.29	0.82	0.54	0.79	0.59	0.70 (0.56-0.84)
**GAS6 + AXL + TYRO3 + MERTK**		0.69	0.79	0.88	0.54	0.82 (0.72-0.92)
**AXL + TYRO3**		0.71	0.79	0.88	0.56	0.81(0.71-0.91)
**AXL + GAS6**		0.55	0.88	0.90	0.48	0.76 (0.64-0.87)
**AXL + MERTK**		0.57	0.92	0.94	0.50	0.76 (0.65-0.88)
**TYRO3 + GAS6**		0.59	0.88	0.91	0.50	0.79 (0.68-0.90)
**TYRO3 + MERTK**		0.96	0.46	0.79	0.85	0.77 (0.66-0.88)
**MERTK + GAS6**		0.98	0.50	0.81	0.92	0.74 (0.61-0.87)
**AXL + TYRO3 + GAS6**		0.71	0.79	0.88	0.56	0.81 (0.71-0.91)
**AXL + TYRO3 + MERTK**		0.69	0.83	0.90	0.56	0.82 (0.72-0.92)
**AXL + GAS6 + MERTK**		0.67	0.79	0.87	0.53	0.76 (0.64-0.87)
**TYRO3 + GAS6 + MERTK**		0.80	0.63	0.82	0.60	0.79 (0.68-0.90)

AUC area under the curve; CI confidence interval; PPV positive predictive value; NPV negative predictive value

Supplementary table S4. Screening efficacy of individual and combined salivary TAM biomarkers between periodontal health and mild periodontitis (stages I/II).

Biomarker	Cut off (pg/mL)	Sensitivity	Specificity	PPV	NPV	AUC (95% CI)
**GAS6**	172.66	0.88	0.68	0.73	0.85	0.83 (0.72-0.94)
**AXL**	321.435	0.64	0.96	0.94	0.73	0.83 (0.71-0.94)
**TYRO3**	633.66	0.76	0.80	0.79	0.77	0.80 (0.68-0.92)
**MERTK**	116.42	0.44	0.84	0.73	0.60	0.59 (0.43-0.76)
**GAS6 + AXL + TYRO3 + MERTK**		0.84	0.84	0.84	0.84	0.90 (0.81-0.98)
**AXL + TYRO3**		0.80	0.92	0.91	0.82	0.89 (0.79-0.98)
**AXL + GAS6**		0.84	0.80	0.81	0.83	0.87 (0.77-0.97)
**AXL + MERTK**		0.68	0.92	0.89	0.74	0.83 (0.71-0.95)
**TYRO3 + GAS6**		0.76	0.84	0.83	0.78	0.85 (0.74-0.96)
**TYRO3 + MERTK**		0.76	0.80	0.79	0.77	0.80 (0.67-0.92)
**MERTK + GAS6**		0.68	0.84	0.81	0.72	0.83 (0.72-0.94)
**AXL + TYRO3 + GAS6**		0.80	0.92	0.91	0.82	0.89 (0.81-0.98)
**AXL + TYRO3 + MERTK**		0.76	0.92	0.90	0.79	0.90 (0.81-0.98)
**AXL + GAS6 + MERTK**		0.84	0.88	0.88	0.85	0.88 (0.78-0.97)
**TYRO3 + GAS6 + MERTK**		0.76	0.84	0.83	0.78	0.85 (0.75-0.96)

AUC area under the curve; CI confidence interval; PPV positive predictive value; NPV negative predictive value

Supplementary table S5. Screening efficacy of individual and combined salivary TAM biomarkers between periodontal health and severe periodontitis (stages III/IV).

Biomarker	Cut off (pg/mL)	Sensitivity	Specificity	PPV	NPV	AUC (95% CI)
**GAS6**	353.89	0.54	0.96	0.93	0.67	0.83 (0.73-0.94)
**AXL**	321.44	0.69	0.96	0.95	0.75	0.88 (0.79-0.97)
**TYRO3**	912.20	0.65	0.96	0.94	0.73	0.89 (0.81-0.98)
**MERTK**	91.76	0.69	0.72	0.72	0.69	0.72 (0.57-0.86)
**GAS6 + AXL + TYRO3 + MERTK**		0.69	1	1	0.76	0.91 (0.84-0.99)
**AXL + TYRO3**		0.69	1	1	0.76	0.90 (0.82-0.98)
**AXL + GAS6**		0.69	0.92	0.90	0.74	0.87 (0.77-0.96)
**AXL + MERTK**		0.65	0.96	0.94	0.73	0.89 (0.81-0.97)
**TYRO3 + GAS6**		0.69	0.96	0.95	0.75	0.90 (0.83-0.98)
**TYRO3 + MERTK**		0.65	0.96	0.94	0.73	0.88 (0.79-0.97)
**MERTK + GAS6**		0.54	0.96	0.93	0.67	0.84 (0.73-0.94)
**AXL + TYRO3 + GAS6**		0.69	1	1	0.76	0.90 (0.82-0.98)
**AXL + TYRO3 + MERTK**		0.69	1	1	0.76	0.91 (0.84-0.99)
**AXL + GAS6 + MERTK**		0.69	0.96	0.95	0.75	0.88 (0.80-0.97)
**TYRO3 + GAS6 + MERTK**		0.69	0.96	0.95	0.75	0.9 (0.82-0.98)

AUC area under the curve; CI confidence interval; PPV positive predictive value; NPV negative predictive value

Supplementary table S6. Screening efficacy of individual and combined salivary TAM biomarkers between gingivitis and mild periodontitis (stages I/II).

Biomarker	Cut off (pg/mL)	Sensitivity	Specificity	PPV	NPV	AUC (95% CI)
**GAS6**	172.23	0.88	0.54	0.67	0.81	0.73 (0.58-0.87)
**AXL**	402.80	0.56	0.92	0.88	0.67	0.74 (0.60-0.88)
**TYRO3**	649.35	0.76	0.67	0.70	0.73	0.72 (0.58-0.87)
**MERTK**	40.65	0.96	0.34	0.62	0.90	0.66 (0.50-0.81)
**GAS6 + AXL + TYRO3 + MERTK**		0.64	0.79	0.76	0.68	0.78 (0.65-0.91)
**AXL + TYRO3**		0.80	0.71	0.74	0.77	0.78 (0.65-0.91)
**AXL + GAS6**		0.64	0.75	0.73	0.67	0.74 (0.60-0.88)
**AXL + MERTK**		0.56	0.92	0.88	0.67	0.74 (0.60-0.88)
**TYRO3 + GAS6**		0.72	0.71	0.72	0.71	0.75 (0.62-0.89)
**TYRO3 + MERTK**		0.76	0.63	0.68	0.71	0.71 (0.56-0.86)
**MERTK + GAS6**		1	0.5	0.68	1	0.74 (0.59-0.88)
**AXL + TYRO3 + GAS6**		0.64	0.79	0.76	0.68	0.78 (0.65-0.91)
**AXL + TYRO3 + MERTK**		0.80	0.71	0.74	0.77	0.78 (0.64-0.91)
**AXL + GAS6 + MERTK**		0.80	0.58	0.67	0.74	0.74 (0.60-0.88)
**TYRO3 + GAS6 + MERTK**		0.72	0.71	0.72	0.71	0.76 (0.62-0.89)

AUC area under the curve; CI confidence interval; PPV positive predictive value; NPV negative predictive value

Supplementary table S7. Screening efficacy of individual and combined salivary TAM biomarkers between gingivitis and severe periodontitis (stages III/IV).

Biomarker	Cut off (pg/mL)	Sensitivity	Specificity	PPV	NPV	AUC (95% CI)
**GAS6**	134.08	0.92	0.5	0.67	0.86	0.75 (0.61-0.89)
**AXL**	378.04	0.58	0.92	0.88	0.67	0.77 (0.65-0.90)
**TYRO3**	353.3	1	0.5	0.68	1	0.82 (0.70-0.93)
**MERTK**	57.18	1	0.5	0.68	1	0.73 (0.59-0.88)
**GAS6 + AXL + TYRO3 + MERTK**		0.65	0.96	0.94	0.72	0.84 (0.73-0.95)
**AXL + TYRO3**		0.62	0.96	0.94	0.70	0.83 (0.72-0.94)
**AXL + GAS6**		0.54	0.92	0.88	0.65	0.77 (0.64-0.90)
**AXL + MERTK**		0.65	0.88	0.85	0.70	0.79 (0.66-0.91)
**TYRO3 + GAS6**		0.62	0.92	0.89	0.69	0.83 (0.72-0.94)
**TYRO3 + MERTK**		0.58	0.92	0.88	0.67	0.82 (0.71-0.93)
**MERTK + GAS6**		0.88	0.50	0.66	0.80	0.75 (0.61-0.88)
**AXL + TYRO3 + GAS6**		0.65	0.96	0.94	0.72	0.84 (0.73-0.95)
**AXL + TYRO3 + MERTK**		0.62	0.96	0.94	0.70	0.85 (0.74-0.95)
**AXL + GAS6 + MERTK**		0.65	0.83	0.81	0.69	0.78 (0.65-0.91)
**TYRO3 + GAS6 + MERTK**		0.62	0.92	0.89	0.69	0.83 (0.72-0.94)

AUC area under the curve; CI confidence interval; PPV positive predictive value; NPV negative predictive value
